# A Collision Tumour Phenomenon: Dual Metastatic Deposits From Melanoma and Adrenal Carcinoma Causing Small Bowel Intussusception

**DOI:** 10.7759/cureus.114302

**Published:** 2026-08-10

**Authors:** Mikaella Toquero, Joseph Waddon, Hussam K Mohamed, Charlotte Hawkins, Palanichamy Chandran

**Affiliations:** 1 General Surgery, Wrexham Maelor Hospital, Wrexham, GBR

**Keywords:** adrenal carcinoma, adult intussusception, bowel obstruction, collision tumour, jejunal intussusception, malignant melanoma, small bowel intussusception, small bowel resection

## Abstract

Adult intussusception is rare and is usually associated with an underlying pathological lead point, often malignancy. While melanoma commonly metastasises to the gastrointestinal tract, melanoma-related intussusception remains uncommon, and small bowel metastasis from adrenal cortical carcinoma is exceptionally rare. This case describes synchronous metastatic melanoma and adrenal carcinoma causing jejunal intussusception. A 43-year-old male with stage 3B melanoma and an oligometastatic right adrenal lesion presented with two days of bilious vomiting and reduced oral intake, with minimal abdominal symptoms and no significant examination findings. CT imaging, prompted by his oncological history, revealed high-grade small bowel obstruction secondary to jejunal intussusception. He underwent laparoscopic-assisted reduction and small bowel resection, with a 5 cm melanoma deposit identified as the lead point. Histopathology demonstrated a rare collision tumour comprising metastatic melanoma and metastatic adrenal cortical carcinoma. The patient recovered well postoperatively and was discharged home. This case highlights the diagnostic challenge of malignant adult intussusception, particularly in patients with complex oncological histories and subtle clinical presentations. The coexistence of metastatic melanoma and adrenal cortical carcinoma as dual pathological lead points represents an exceptionally rare finding. It emphasises the importance of maintaining clinical suspicion, early cross-sectional imaging and multidisciplinary management in oncology patients presenting with gastrointestinal symptoms. Synchronous metastatic melanoma and adrenal cortical carcinoma causing small bowel intussusception is an exceptionally rare presentation. A low threshold for imaging and timely intervention is essential to avoid diagnostic delay and optimise outcomes in patients with metastatic malignancy.

## Introduction

Adult intussusception is an uncommon clinical entity, representing only 1% of small bowel obstructions in adults and typically arising secondary to an underlying benign or malignant lead point, with metastatic melanoma recognised as a rare but well-documented cause [1-3]. Unlike paediatric intussusception, which is typically idiopathic, up to 90% of adult cases have an identifiable anatomical lead point and approximately half are malignant [2,4].

The small bowel is a recognised site for metastatic disease, particularly for melanoma, which demonstrates a strong tendency for gastrointestinal spread via lymphatic and haematogenous routes; this most commonly spreads to the small bowel in advanced disease [3,5,6,7]. Small bowel metastases are frequently clinically silent or present with non-specific symptoms such as anaemia, weight loss, or intermittent abdominal discomfort [6,7]. When symptomatic, they may result in complications including bleeding, obstruction or intussusception secondary to a pathological lead point [5,7].

Despite this recognised predisposition for gastrointestinal spread, intussusception secondary to metastatic melanoma remains an unusual presentation, described only in isolated case reports and small case series, and is only rarely reported to act as a pathological lead point [1,3,5,6].

Adrenocortical carcinoma, by contrast, is an aggressive but rare malignancy that typically metastasises to the liver, lungs, and lymph nodes, with small bowel involvement being distinctly uncommon [8]. Such lesions may only rarely be reported to act as a pathological lead point for intussusception [5,9]. The concurrent presence of two distinct metastatic deposits, from separate primary malignancies, acting simultaneously as lead points for intussusception represents an exceptionally rare phenomenon. A PubMed search (collision AND melanoma AND adrenal carcinoma) identified no previously reported cases of synchronous metastatic melanoma and adrenocortical carcinoma causing small bowel intussusception [5,7,9,10]. This may represent the clinical significance and novelty of this presentation.

Collision tumours are uncommon entities in which two or more histologically distinct neoplasms arise adjacent to one another at the same anatomical site, coexisting with little or no intermingling between them [11]. This distinguishes them from composite tumours, which involve two morphologically distinct components arising from a common neoplastic source rather than two independently arising tumours; It also distinguishes them from synchronous or multifocal disease, in which lesions occur independently at separate, non-contiguous anatomical sites [12]. The pathogenesis of collision tumours remains poorly understood, with coincidental, independent tumorigenesis at a shared site considered the most likely explanation.

This case highlights an unusual and diagnostically challenging scenario in which two distinct metastatic malignancies simultaneously contribute to jejunal intussusception, which could be considered a collision tumour. In addition, this case presented with minimal abdominal findings on presentation. This emphasises the need for heightened clinical suspicion, careful radiological interpretation and timely surgical intervention in patients with complex oncological histories.

## Case presentation

A 43-year-old male presented to the emergency department with a two-day history of bilious, non-projectile vomiting, occurring 6-8 times per day, accompanied by markedly reduced oral intake. He reported mild, intermittent, and generalised abdominal discomfort without focal pain, which resolved with simple analgesia. On arrival to the hospital, he was pain-free with his predominant symptom being persistent nausea and vomiting. He continued to pass flatus, and his last bowel movement two days earlier was consistent with his baseline habit. He denied weight loss, gastrointestinal bleeding, urinary symptoms, or respiratory complaints. 

His past medical history included Stage 3B malignant melanoma of the back diagnosed in February 2025, an oligometastatic right adrenal gland lesion diagnosed in March 2026, and asthma. He had finished undergoing systemic immunotherapy of pembrolizumab for the melanoma and had completed his seventh cycle; the timeframe of this treatment was from April 2025 to April 2026 and was discontinued due to disease progression. His last treatment was six weeks prior to admission. 

In terms of social history, he lived with his wife and worked as a heavy goods vehicle (HGV) driver. He was a non-smoker and denied alcohol consumption or recreational drug use. No recent travel history, dietary changes or infectious contacts were reported. 

On presentation, the patient’s observations were taken, and he was haemodynamically stable, as shown in Table 1. 

**Table 1:** Observation at the time of presentation to the emergency department

Parameter	Value
Blood pressure	124/83 mmHg
Heart rate	94 bpm
SpO2	99% on room air
Respiratory rate	17 breaths/min
Temperature	37 degrees celsius

Abdominal examination revealed a soft abdomen with mild intermittent generalised discomfort. There was no guarding, rebound tenderness, or peritonism. Bowel sounds were present. No renal angle tenderness was elicited. Cardiorespiratory and neurological examinations were unremarkable. 

As shown in Table 2, initial blood investigations demonstrated a neutrophilic leucocytosis, thrombocytosis, and an elevated C-reactive protein; this was consistent with an inflammatory response. Mild hypernatraemia, likely reflecting dehydration secondary to prolonged bilious vomiting, was also present. Serum lactate was within the normal range. 

**Table 2:** Bloods investigation on arrival to the emergency department

Investigation	Value	Unit	Reference range
Full blood count			
Haemoglobin (Hb)	104	g/L	120-160
Mean corpuscular volume (MCV)	91	fL	80-100
Platelets (Plt)	736	x10⁹/L	150-400
White cell count (WCC)	15.3	x10⁹/L	4.0-11.0
Neutrophils	12.3	x10⁹/L	1.8-7.5
Inflammatory markers			
C-reactive protein (CRP)	59	mg/L	<5
Urea and electrolytes			
Sodium	149	mmol/L	135-145
Potassium	4.7	mmol/L	3.5-5.0
Urea	5.7	mmol/L	2.5-7.8
Creatinine	82	µmol/L	60-110
eGFR	89	mL/min/1.73m2	>60
Liver function tests			
Bilirubin	8	µmol/L	<21
Albumin	38	g/L	35-50
Alkaline phosphatase (ALP)	169	U/L	30-130
Alanine aminotransferase (ALT)	40	U/L	10-49
Other			
Lactate	1.6	mmol/L	<2.0

The provisional working diagnosis at this stage was viral gastroenteritis, given the unremarkable abdominal examination and pain-free status. A CT abdomen and pelvis was subsequently arranged as per the request of the patient's oncologist, to assess for any treatment-related intra-abdominal pathology or any disease progression from his well-known cancer background. This scan occurred on day 2 of admission. 

**Figure 1:**
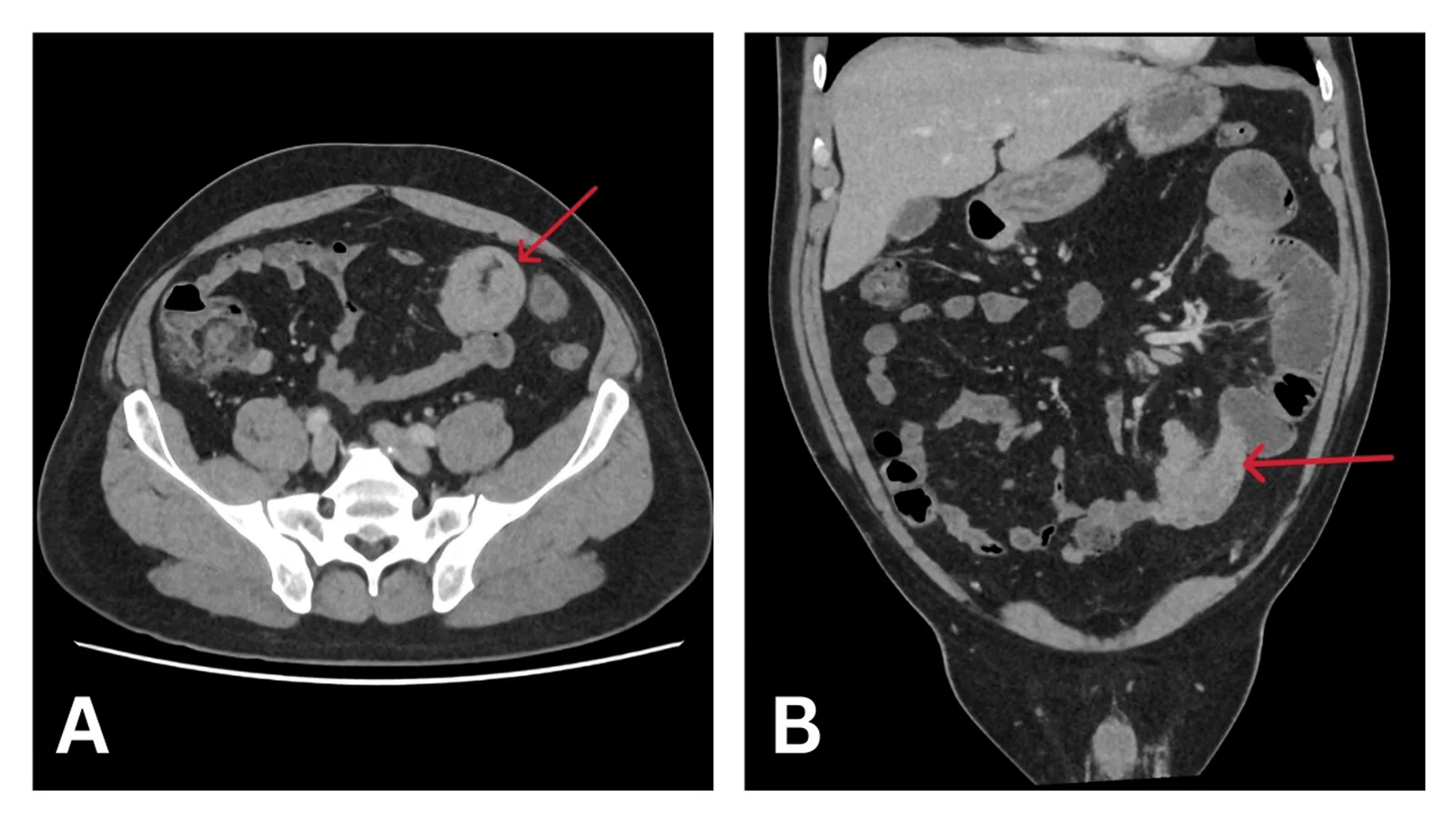
A: Axial CT image demonstrating a well-defined soft tissue mass (arrow) with a central mesenteric fat/vessel component, in keeping with a target sign of small bowel intussusception. B: Coronal oblique reconstruction demonstrating the bowel within the bowel sign of small bowel intussusception (arrow), corresponding to the longitudinal appearance of the intussuscepted bowel segment.

A CT abdomen and pelvis showed high-grade small bowel obstruction secondary to intussusception with features suggestive of disease progression from both melanoma and right adrenal carcinoma; however, whether this was true progression or pseudo-progression was not determinable. The images demonstrated a well-defined, layered soft tissue mass within a loop of small bowel in the left upper quadrant, producing a "target" or "sausage-shaped" configuration in axial section (Figure 1A); this is in keeping with an intussusception. 

Coronal oblique images (Figure 1B) confirmed the longitudinal extent of the intussusception, with the intussusceptum and its associated mesenteric fat and vessels seen telescoping into the distal intussuscipiens, producing the "bowel-within-bowel" appearance. 

The patient was prepared and listed for emergency bowel resection and anastomosis. He received a transfusion consisting of one unit of RBC, intravenous iron and a unit of human albumin infusion for further pre-operative optimisation. 

Laparoscopically assisted intussusception reduction and small bowel resection were done on day 3 of admission. This proved an intussuscepting melanoma deposit in the small bowel, causing obstruction in the left iliac fossa region. The segment of small bowel resected was 400 mm in length with a diameter of 40 mm. A constricting lesion was noted 55 mm from the nearest resection margin; after further serial sectioning, a black loculated solid lesion was noted in the lumen (45 x 40 x 20 mm). 

The small bowel wall was infiltrated by an epithelioid neoplasm involving the submucosa and extending through the muscularis propria into the subserosa. The tumour comprised pleomorphic cells in sheets and nests, with enlarged nuclei, prominent nucleoli and eosinophilic cytoplasm; mitotic figures were readily identified. Coarse brown intracellular and extracellular pigment was present. The overlying mucosa was ulcerated with inflammation. These features are in keeping with metastatic malignant melanoma. 

**Figure 2:**
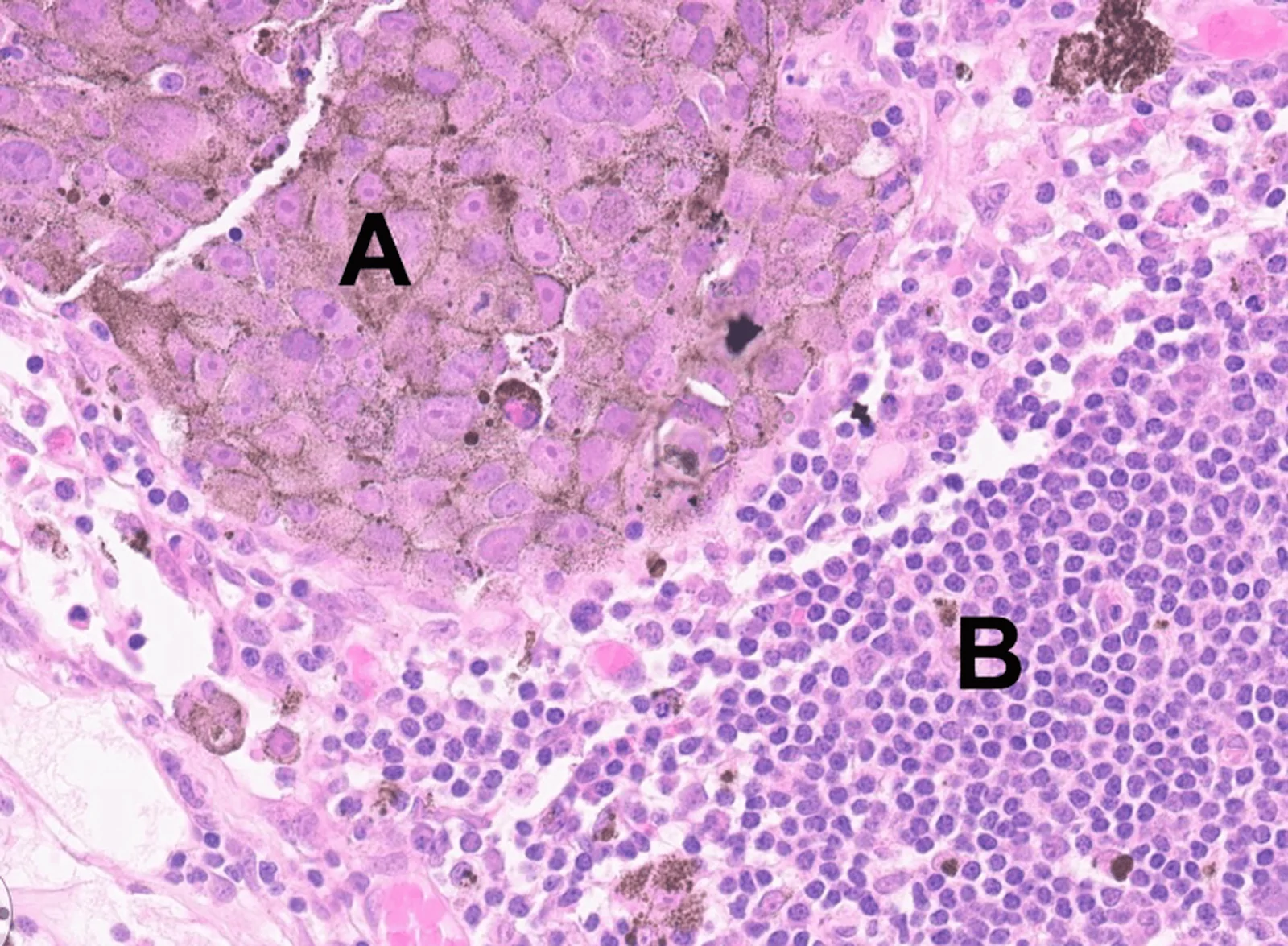
Collision tumour within the small bowel wall (H&E, ×20). (A) Pigmented epithelioid cells with intracytoplasmic melanin, consistent with metastatic melanoma. (B) Adjacent basophilic small cells with high N:C ratio, consistent with metastatic adrenal carcinoma. H&E: haematoxylin and eosin stain; x20: times 20 magnification of the original histological size;  N:C ratio: nuclear-to-cytoplasmic ratio

The small bowel wall was infiltrated by a biphasic malignant process with a similar pattern of invasion. One component (Figure 2A) corresponded to the melanoma population described above. This population was in direct apposition, without an intervening capsule, to a second, histologically distinct population (Figure 2B) composed of smaller, monomorphic basophilic cells with high nuclear-to-cytoplasmic ratios and scant cytoplasm. These features are in keeping with a collision tumour comprising metastatic malignant melanoma (Figure 2A) and a second metastatic malignancy (Figure 2B), morphologically consistent with adrenal carcinoma. 

Postoperatively, the patient recovered very well. His pain was optimised by the pain team, he tolerated eating and drinking well, his bowels opened on day 4 postoperatively, and he was discharged home on day 5 post-surgery. There were no immediate post-operative complications. The patient remained under the care of the oncologist, who scheduled follow-up to discuss the next steps in treatment options. Due to involvement of a mesenteric lymph node, the patient was no longer suitable for adrenalectomy and was commenced on first-line palliative Opdualag. 

## Discussion

Gastrointestinal metastasis is a recognised feature of advanced melanoma, with the small bowel representing the most frequently affected gastrointestinal site [5-7]. Despite this predilection, clinically apparent small bowel involvement remains uncommon, and many cases are detected incidentally during imaging or identified post‑mortem [3,6]. When symptomatic, metastatic deposits may lead to complications, such as bleeding, obstruction or intussusception, the latter occurring when a tumour acts as a pathological lead point [9,10,13]. 

Adult intussusception is an uncommon cause of bowel obstruction, accounting for approximately 1% of adult cases [4,13]. In contrast to paediatric cases, which are often idiopathic, adult intussusception is typically associated with an identifiable structural lesion; malignancy represents a significant proportion of these lead points [10,13]. Melanoma has a recognised tendency for gastrointestinal metastasis [5-7], particularly involving the small bowel, where metastatic deposits may cause complications including obstruction and intussusception due to their ability to act as pathological lead points [9,14,15]. 

It must be noted that CT imaging played a pivotal role in the diagnosis in this case report. It remains the most sensitive modality for identifying adult intussusception and detecting underlying lead-point lesions [10,16]. In this case, it was crucial in expediting definitive surgical management. 

This case is notable not only for the subtlety of presentation, but also for the fact that the intussusception was caused not by a single malignant lead point; it was caused by dual metastatic deposits from two distinct primary cancers - melanoma and adrenal carcinoma. Both metastatic deposits demonstrated histologically distinct morphology, arising from separate primary malignancies within the same jejunal segment without intermixing, findings suggestive of a true collision tumour rather than a single heterogeneous metastatic lesion. As immunohistochemical staining was not performed, this diagnosis is based on morphological assessment alone. IHC may have provided further confirmatory evidence of the distinct cellular origins of each component, and its absence is acknowledged as a limitation of this report. 

Adrenal cortical carcinoma rarely metastasises to the small bowel and synchronous metastatic involvement with melanoma is extraordinarily uncommon. The coexistence of two separate malignant lesions within the same jejunal segment complicates both diagnosis and interpretation of imaging. Notably, pembrolizumab (started in April 2025) was discontinued due to progression in April 2026, with intussusception following a month later; this possibly reflects differential control, with the melanoma suppressed while the less immunotherapy-responsive adrenal component progressed unchecked. This underscores the need for heightened suspicion in patients with complex oncological histories, particularly when progression on immunotherapy may signal an unrecognised second malignancy rather than the treated primary. 

As previously stated, the clinical presentation in this case was deceptively vague. Despite significant underlying pathology requiring surgical management, the patient presented with isolated vomiting and mild abdominal discomfort, without peritonitis or overt obstructive signs. This initially suggested a non-surgical diagnosis. This depicts the limited sensitivity of clinical assessment in high-risk oncology patients and reinforces the importance of early cross-sectional imaging. 

## Conclusions

This case demonstrates an atypically subtle presentation of a significant mechanical bowel obstruction, in addition to findings suggesting an exceptionally rare collision tumour phenomenon, in which synchronous metastatic melanoma and adrenal cortical carcinoma produced a pathological lead point resulting in small bowel intussusception. 

The subtle clinical presentation highlights how a low threshold for CT imaging in patients with cancer and complex oncological histories with persistent gastrointestinal symptoms should be adopted, even when abdominal examination findings are reassuring. Early CT imaging and multidisciplinary involvement are essential to avoid diagnostic delay and optimise outcomes in patients with complex metastatic disease.
